# Induction of Neural Differentiation and Protection by a Novel Slow-Release Nanoparticle Estrogen Construct in a Rat Model of Spinal Cord Injury

**DOI:** 10.1007/s11064-024-04289-4

**Published:** 2024-11-30

**Authors:** Azizul Haque, Vandana Zaman, Kelsey P. Drasites, Denise Matzelle, Sushant Sawant, Alexey Vertegel, Abhay Varma, Naren L. Banik

**Affiliations:** 1 Department of Microbiology and Immunology, Medical University of South Carolina, 173 Ashley Avenue, Charleston, SC 29425 USA; 2 Department of Neurosurgery, Medical University of South Carolina, 96 Jonathan Lucas Street, Charleston, SC 29425 USA; 3 Ralph H. Johnson Veterans Administration Medical Center, 109 Bee St, Charleston, SC 29401 USA; 4 Department of Bioengineering, Clemson University, Clemson, SC USA

**Keywords:** Spinal cord injury, Slow-release nanoparticle-estrogen, Gliosis, Myelination, Neural differentiation, Bladder function, Locomotor function

## Abstract

Spinal cord injury (SCI) is a complex debilitating condition leading to permanent life-long neurological deficits. Estrogen (E2) treatment is known to be neuroprotectant in SCI. This hormone is highly pleiotropic and has been shown to decrease apoptosis, modulate calcium signaling, regulate growth factor expression, act as an anti-inflammatory, and drive angiogenesis. These beneficial effects were found in our earlier study at the low dose of 10 µg/kg E2 in rats. However, the dose remains non-physiologic, which poses a safety hurdle for clinical use. Thus, we recently devised/constructed a fast release nanoparticle (NP) estrogen embedded (FNP-E2) construct and tested a focal delivery system in a contused SCI rat model which showed protection in the short run. In the current study, we have developed a novel slow-release NP estrogen (SNP-E2) delivery system that shows sustained release of E2 in the injured spinal cord and no systemic exposure in the host. The study of E2 release and kinetics of this SNP-E2 construct in vitro and in vivo supported this claim. Delivery of E2 to the injured spinal cord via this approach reduced inflammation and gliosis, and induced microglial differentiation of M1 to M2 in rats after SCI. Analysis of spinal cord samples showed improved myelination and survival signals (AKT) as demonstrated by western blot analysis. SNP-E2 treatment also induced astrocytic differentiation into neuron-like (MAP2/NeuN) cells, supported the survival of oligodendrocyte precursor cells (OPC), and improved bladder and locomotor function in rats following SCI. These data suggest that this novel delivery strategy of SNP-E2 to the injured spinal cord may provide a safe and effective therapeutic approach to treat individuals suffering from SCI.

## Introduction

Spinal cord injury (SCI) can result in severe neurological deficits such as loss of sphincter function, paralysis, and neurogenic muscle loss due to immobility [1–4]. The primary injury in SCI is an instantaneous, irreversible necrotic cell death at the injury site, but the secondary injury and ischemic cell death that occurs in neighboring tissue (penumbra) may be reversible if treated early [4–6]. Following SCI, local inflammation and infiltration of inflammatory cells produce factors such as cytokines/chemokines, production of reactive oxygen species (ROS), activation of calpain, glial scarring, etc.; these destroy tissue and prevent axonal regeneration and recovery of locomotor function [4, 7]. Failure of the CNS to repair and regenerate following an injury has also been associated with the decreased ability of macrophage/microglia to properly transition from M1 to M2 types [8–10]. Similarly, astrocytic differentiation and neuronal-astroglial interactions can potentially occur, influencing the survival of axons and neurons. Currently, no FDA approved drug is available to effectively treat SCI due to the complex pathophysiology involved. Although methylprednisolone was widely used historically in acute injury, its use remains controversial due to multiple side effects [11–13]. Since the destructive process in SCI is multifactorial [5], developing an effective therapeutic agent to attenuate tissue damage and improve function following injury is imperative.

Our laboratory was among the first to show the steroid hormone estrogen (E2, 17β-estradiol) mediated neuroprotection and improved locomotor function in experimental SCI in rats [14, 15]. E2 is a highly pleotropic agent that activates transcription of E2-regulated genes; it is an anti-inflammatory, antioxidant, and anti-apoptotic agent that reduces gliosis and has neurotrophic and angiogenic properties [16–18]. As an anti-inflammatory agent, E2 drives M2 polarization of microglial cells in experimental models of hypoxia, favoring regeneration [19]. Our recent study with a fast-release nanoparticle (NP) estrogen (FNP-E2) suggests that it decreases CSPG and glial fibrillary acidic protein (GFAP) expression in astrocytes and reduces proliferation of microglia in acute SCI [20]. Thus, focal delivery of E2 to the spinal cord may attenuate activation and proliferation of microglia and astrocytes, thereby diminishing glial scar and axonal damage in SCI.

E2 has exhibited a neuroprotective effect not only in SCI models [21, 22] but also in experimental traumatic brain injury [23] and stroke [24], suggesting E2 warrants clinical evaluation in neurotrauma. However, the FDA-approved drug Premarin (conjugated estrogens containing 8–10% β-estradiol) has significant safety concerns [25]. Traditional systemic dosing (oral, i.v.) of E2 poses a hurdle for use in SCI individuals who are already at an elevated risk for deep venous thromboembolism (DVT) as short term E2 treatment used for contraception is associated with increased risks of DVT [26]. To minimize risk and maximize function, investigations of pharmacodynamics of E2 (1–200 µg/g body weight) in dose-dependent and time-dependent studies revealed neuroprotective effects of low dose E2 (5–10 µg) with improved locomotor function in rat SCI [27, 28].

Focally delivering E2 to the injured spinal cord via NPs may help achieve maximal efficacy while keeping systemic levels of E2 in a physiological range to avoid toxicity. We have recently formulated a novel strategy for fast and slow release E2 delivery with the hope that the acute injury in SCI will be attenuated by direct delivery of fast-release E2 to the injured spinal cord, and then a slow-release delivery of FNP-E2 to have sustained E2 concentrations in the injured spinal cord. While this study is focused on the slow-release nanoparticle (NP) estrogen (SNP-E2) construct, the overall goal is to co-administer the slow-release nano-E2 (SNP-E2) which may increase availability of E2 and prolong the therapeutic effects of FNP-E2 to decrease inflammation in subacute and chronic phases of the SCI. Interestingly, our recent studies with FNP-E2 have shown decreased post-injury lesion size, reactive gliosis, and glial scar formation in acute phases of SCI [20, 29]. Our results also showed increased axonal regeneration, vascular endothelial growth factor production, and glial-cell-derived neurotrophic factor(s) following FNP-E2 administration in SCI rats. Concomitantly improved locomotor and bladder functional recovery was observed with FNP-E2 treatment [29]. Therefore, low-dose site-directed estrogen delivery may provide a therapeutic approach for enhanced axonal/neuronal repair and functional recovery in SCI individuals.

In addition, we have recently shown that focal delivery of E2 via nanoparticles (NPs), fast release NP estrogen (FNP-E2), increased tissue distribution of E2 over time, attenuated cell death, and improved myelin preservation in injured spinal cord [30]. Specifically, the FNP-E2 construct reduced the Bax/Bcl-2 ratio in injured spinal cord tissues, and reduced gliosis and penumbral demyelination distal to the lesion site [30]. While the FNP-E2 construct decreased gliosis and improved locomotor function, sustained recovery and improvement of function may require the use of a slow-release NP estrogen (SNP-E2) construct. This is particularly important because remyelination and functional recovery in SCI take time. Thus, an SNP-E2 construct has been devised to investigate whether sustained delivery of E2 will impact glial and neuronal responses and improve myelination following SCI. This study aims to determine whether targeted delivery of SNP-E2 will lead to neuroprotection and subsequent improvement of locomotor functional recovery. SNP-E2 delivered for a prolonged period of time allowed for extended bioavailability to increase the therapeutic window of E2 (and transition from acute to subacute SCI treatment), enhanced regeneration, and improved function.

## Materials and Methods

### Induction of SCI

Adult male Sprague-Dawley rats (200–250 g) were used for the induction of SCI and treatment with slow-release NP-encapsulated E2. While gender-related differences may be found in SCI where females are favored in terms of tissue preservation and locomotor recovery [31], no significant differences between males and females could be seen in SCI regarding the localization, onset, or distribution of pain. Moreover, factors affecting number of painful body regions, pain descriptors, ratings of pain intensities, and life satisfaction were not significantly different. Thus, male rats were used in this study of SCI. Rats were anesthetized with ketamine (80 mg/kg)/xylazine (10 mg/kg). Following anesthetization of the animal and preparation of the surgical site, a midline incision was made on the back over the spinous processes, and a T-10 laminectomy was performed. SCI induction was performed as previously described [32–34]. Briefly, the spine was immobilized with a stereotactic device, and the injury was induced via the method of Perot: dropping a constant weight (5 g) from a height of 8 cm onto an impounder (0.3 cm in diameter) gently placed on the spinal cord.

Vehicle-treated animals received the same volume of void NPs in buffer (100 µL). Sham animals underwent a T-10 laminectomy [27, 32, 35]. Wounds were closed using 5 − 0 absorbable suture (for interior wound closure). Exterior wound closure was performed with either 4 − 0 ethilon sutures or application of staples 5–8 mm apart (EZ-Clip wound clips). The surgical site was monitored, and based on the rate of healing, sutures are removed under isoflurane anesthesia.

After surgery, animals were placed on a warming pad until awake. Food was placed on the floor of the cage, and long-stemmed water bottles were provided for ease of access to water. Animals were checked twice daily until sacrifice. Animals were visually inspected to check for abnormal behaviors (head pressing, piloerection, hunching) and handled gently for closer physical inspection, including checking for bladder function. The animals’ bladders were monitored and expressed twice daily. Rodents that develop urine scald were bathed with warm water and gently dried. Vaseline was applied to the affected area. Urine was expressed onto a clear glass dish in order to visually check for cloudiness.

If the subjects were not recovering appropriately, monitoring was amplified commensurate with the animal’s health needs. Affected animals were thus treated with antibiotics or analgesics, given soft food, or placed on a heating pad, depending on the symptoms. Blood and tissue samples were collected 7–14 days post-injury. All animal experiments were performed in accordance with the *Guide for the Care and Use of Laboratory Animals* of the US Department of Health and Human Services (National Institutes of Health, Bethesda, MD, USA) and were approved by the Institutional Animal Care and Use Committee (IACUC) at the Medical University of South Carolina under the protocol ARC #1254.

### Preparation of E2-Loaded Slow-Release NPs

NPs were formulated in the Bioengineering Department of Clemson University, South Carolina using the nanoprecipitation method previously described [36–38]. Polylactic acid (PLA) NPs were considered for this study as they are frequently used as nanomedicines, which have advantages over metallic NPs such as the ability to maintain therapeutic drug levels for sustained periods of time. E2-loaded PLA NPs were prepared according to the revised protocol [37]. 5 mg of PLA: PEG (polyethylene glycol), and 5 mg of E2 were dissolved in 1 mL of acetone. The resulting acetone solution was then added dropwise to 20 mL of deionized water, followed by ultrasonication in a bath sonicator (5510 Bransonic^®^ Tabletop cleaner, Branson, Danbury, CT) for 30 min. To remove reagent residues, the obtained samples were centrifuged three times at 7,000 RCF for 2 h and rinsed with PBS. In the case of PLA NPs, 20 mg of pure PLA were used instead of 50:50 PLGA co-polymer. Each batch was evaluated for particle size (poly(lactic-co-glycolic acid), load efficiency, and zeta potential to ensure consistency among batches. After preparation, NPs were stored in sucrose at -20 °C. Unloaded NPs were also synthesized and used as a reference or control. PLA NPs, due to their higher hydrophobicity and slower degradation rate, have been previously shown to have slower drug release as described [38]. Cell viability and proliferation are not significantly altered by PLA-NP as reported [39].

### Gel Plug Delivery System

Gel plugs (0.6% SeaPlaque agarose in PBS) were made by dissolving lyophilized E2-loaded PLA NPs at either 15, 10–5 µg dose (or saline loaded vehicle control) in 50 µL sterile filtered PBS. The gel (final volume 50 uL) was set in PCR amplification tubes overnight to harden. Prior to insertion into animals, gels were sterilized under a tissue culture UV lamp for 15 min as described [29].

### Measurement of E2 Concentrations in Spinal Cord Tissues and Plasma by ELISA

Spinal cord tissue homogenates from PLGA-E2 treatment group were diluted 1:10 − 1:100 in 6% BSA block. Plasma samples from PLA-E2 treatment group were serially diluted and used in the assay. E2 concentration was determined using a commercially available ELISA kit (Calbiotech Estradiol ELISA ES180S) [29]. Undiluted samples were processed following directions provided by the manufacturer. Using protein concentration data gathered from Bradford assay, the tissue sample E2 concentration was then converted from pg/mL to pg/µg of total protein to account for differences in protein concentrations between samples. Plasma E2 concentrations were expressed as pg/ml.

### Western Blot Analysis

Spinal cord caudal sections were homogenized on ice in a standard homogenizing buffer (50 mM Tris-HCl, pH 7.4; 5 mM EGTA; 1 mM phenylmethylsulfonyl fluoride) with protease inhibitor as described [40–42]. Protein concentrations were quantitated and equal amounts of protein (30 µg/lane) from designated samples were separated on a 4–12% Bis/Tris NuPage gel (Invitrogen, Grand Island, NY) [43–45]. Proteins were transferred onto a nitrocellulose membrane (Pierce, Rockford IL), and the blot was probed with MBP (Abcam, ab11159) and AKT (Abcam, ab8805) antibodies. The secondary antibodies used were horseradish peroxidase conjugated anti-mouse (1:2000, Santa Cruz, sc-2005) and anti-rabbit (1:4000, Santa Cruz, sc-2004). A monoclonal antibody for β-actin (1:1000, Santa Cruz, sc-81178) was used as a protein loading control. Relative protein expression was assessed using Image J software (National Institutes of Health, Bethesda, MD) and expressed as relative density for each sample [46–48].

### Immunohistochemistry

Spinal cord tissue segments (lesion and caudal sections) were fixed with 4% paraformaldehyde (PFA) in phosphate-buffered saline (PBS) overnight [34, 35]. Tissue was then embedded in paraffin, sectioned, and mounted onto slides for analysis. Following epitope retrieval, non-specific binding sites were blocked with the serum of the secondary antibody host for 1 h at room temperature. Tissues were incubated with Iba1 (1:250, Abcam ab5076), GFAP (1:250, Santa Cruz sc-9065), iNOS, arginase-1, de-NFP, MBP, O4, and MAP2 antibodies overnight at room temperature. Sections were then incubated with Texas Red^®^ Goat Anti-Rabbit IgG Antibody (Vector Laboratories, Inc., Burlingame, CA), DyLight^®^ 488 Anti-Goat IgG (H + L) (Vector Laboratories, Inc., Burlingame, CA), Texas Red^®^ Anti-Mouse IgM Antibody (Vector Laboratories, Inc., Burlingame, CA), or Fluorescein Anti-Rabbit IgG (Vector Laboratories, Inc., Burlingame, CA) for 1 h in the dark. Slides were mounted with 1 drop of Invitrogen™ ProLong™ Gold Antifade Mountant with DAPI (Thermo Fisher Scientific) and coverslipped [35]. After staining, SCI tissues were viewed under a fluorescence microscope (Nikon A1Rsi Confocal Microscope) with representative images taken at 20× magnification. Using Image J software (National Institutes of Health, Bethesda, MD) the Integrated Fiber Density and Cell numbers were quantified. The image was converted into binary. The region of interest (ROI) was selected, and the cell counts was measured using > Analyze > Analyze Particles. Similarly, the fiber density was measured by converting image into binary and ROI was selected by Edit > Selection > Create Selection.

### Statistical Analysis

Statistical analyses were performed using Microsoft Excel and GraphPad Prism (version 6.0) Software as described [35]. The immunoreactive bands obtained from Western blotting and the immunoreactive pixels of the immunofluorescence data were analyzed with ImageJ software (U.S. National Institutes of Health, Bethesda, MD). Two-tailed paired t-test and one-way ANOVA with Bonferroni test for multiple comparisons were used to determine statistical significance for all other analyses. Data were expressed as mean ± SEM or mean+/STDEV. A p-value < 0.05 was determined to be statistically significant for all calculations.

## Results

### Gel Patch Delivery System and SNP-E2 Bioavailability

**Fig. 1:**
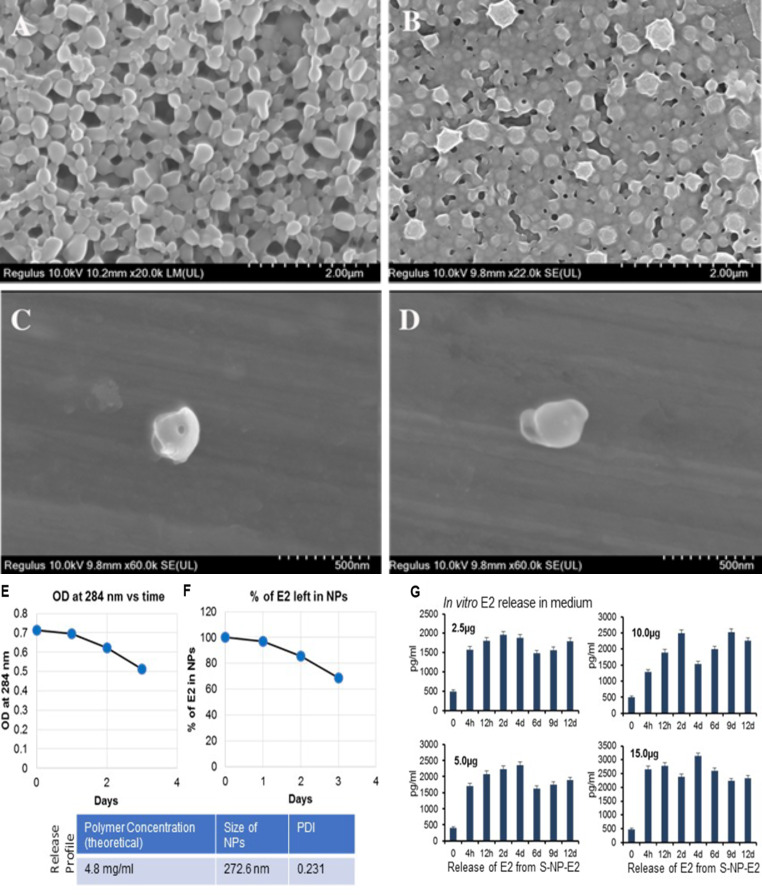
Scanning electron microscope (SEM) images of the estrogen nanoparticles at varying resolutions. (**A** and **C**) Control PLA nanoparticles (SNP) without the estrogen, (**B** and **D**) Estrogen-loaded PLA nanoparticles (SNP-E2). (**E-G**) Availability of E2 from SNP-E2 construct in vitro. Kinetics of E2 released over time. (**E**) %E2 released from SNP-E2 over time, and (**F**) %E2 in NPs over time. Release profile of polymer concentration, size of NPs, and PDI are shown in the insert. (**G**) Kinetics of E2 release in media from four different SNP-E2 constructs (2.5, 5, 10, and 15 µg of E2) in SNP, showing E2 release can be detected until day 12

**Fig. 2:**
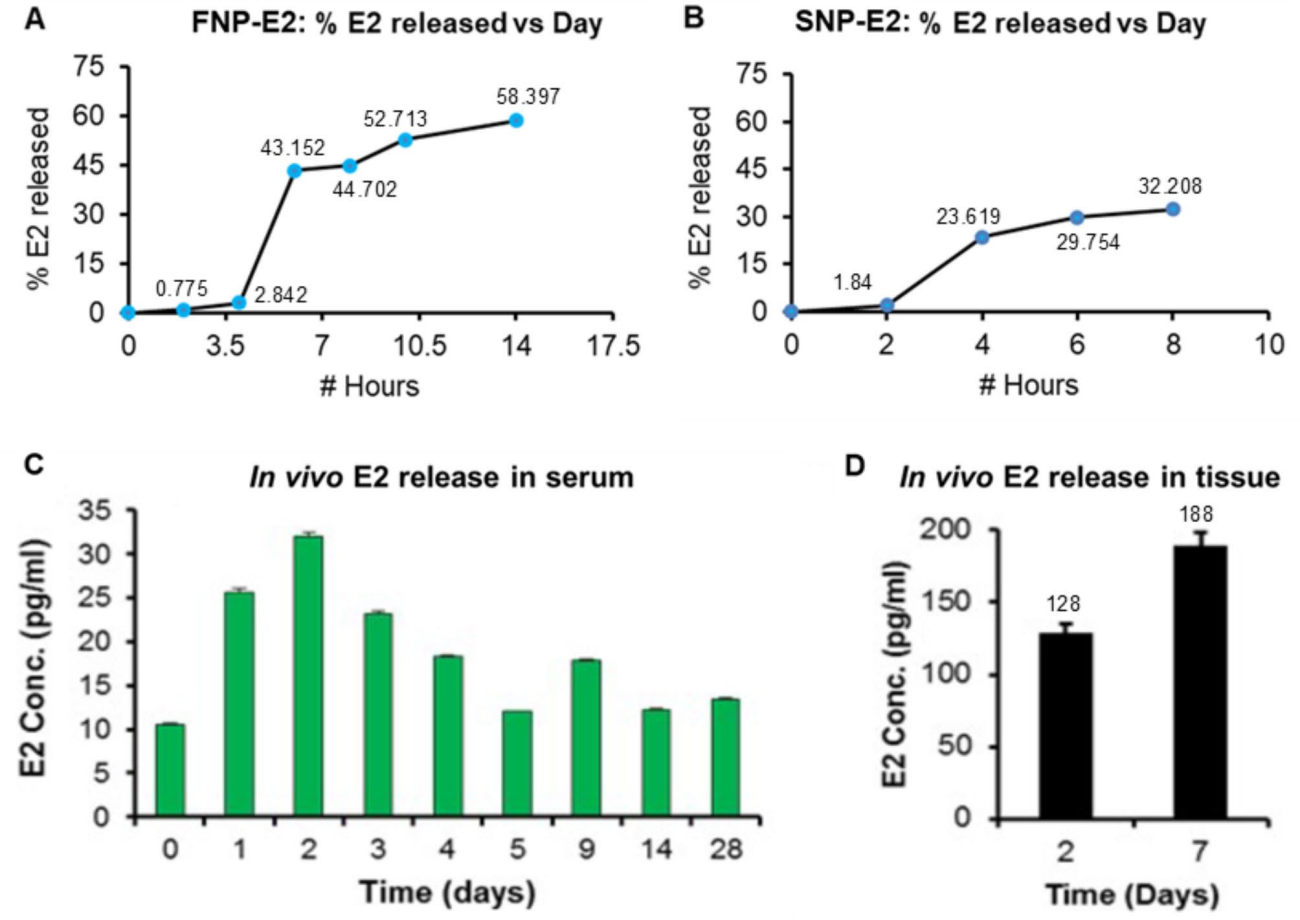
Release of E2 from FNP-E2 and SNP-E2 in vitro and availability of E2 from SNP-E2 in vivo. (**A**) Percent E2 released from FNP-E2 in vitro over time. (**B**) Percent E2 released from SNP-E2 in vitro over time. (**C-D**) Rats were treated with SNP-E2 (5 µg of E2), and serum and spinal cord samples were collected at various time points post SCI. E2 levels in serum (**C**) and spinal cords (**D**) were measured by ELISA (Calbiotech ES180S). Serum E2 concentrations were higher in rats at day 2 and maintained until day 28 post-injury. Tissue E2 at day 7 was also higher as compared to day 2. *n* = 3–4

SNP-E2 was prepared as described in the methods [37, 38] and delivered to the injured spinal cord via a gel patch. Scanning electron microscopy was performed to have images of SNP and SNP-E2 at varying resolutions. Figure 1 shows PLA nanoparticles (SNP) without the Estrogen (A and C), and estrogen-loaded PLA nanoparticles (SNP-E2) (B and D). A moderate to severe (40gcf) SCI was induced by the weight drop method as described [33]. We have previously shown that a fast release FNP-E2 therapy significantly decreases post-SCI lesion size, reactive gliosis, and glial scar formation [29]. The goal of this current study is to provide minimal levels E2 in systemic circulation while providing maximum long-term effects on inflammation, neuronal impairment and functional outcome following SCI. Comparative release of E2 into culture media was tested in vitro, showing kinetics of E2 release from SNP-E2 left in SNP over time (Fig. 1, E-F). Kinetics of E2 release in media was also tested using 2.5, 5, 10, and 15 µg of E2 in SNP (Fig. 1G), suggesting that sustained release of E2 can be detected until day 12. We have previously tested the effects of fast release nanoparticles E2 (FNP-E2) in SCI rats [29]. The current study is focused on SNP-E2. A side-by-side comparison in between FNP-E2 and SNP-E2 regarding their in vitro E2 release kinetics was shown in Fig. 2A and B. The release of E2 from FNP-E2 (Fig. 2A) and SNP-E2 (Fig. 2B) constructs was analyzed at various time points in vitro before testing E2 embedded nanoparticles using a gel patch as described in the methods.

Time course studies of in vivo E2 release in serum showed the release profile at different time point and E2 left in SNP nanoparticles (Fig. 2, C and D). Very low levels of E2 were detected in serum samples until day 28 post-SCI, but tissue concentrations of E2 were slightly elevated at day 7 post-SCI as compared to those detected at day 2 post-SCI (data not shown). E2 release in media was also tested up to day 12. Briefly, SNP-E2 constructs containing 2.5, 5, 10, 15 µg of E2 were tested (Fig. 2E), showing their release pattern in media. Insert shows polymer concentration, size of NPs, PDI, and release profile. These data support the E2 release and feasibility of testing SNP-E2 gel patch approach in the rat SCI model.

### SNP-E2 Elevates AKT and MBP Levels Following SCI

**Fig. 3:**
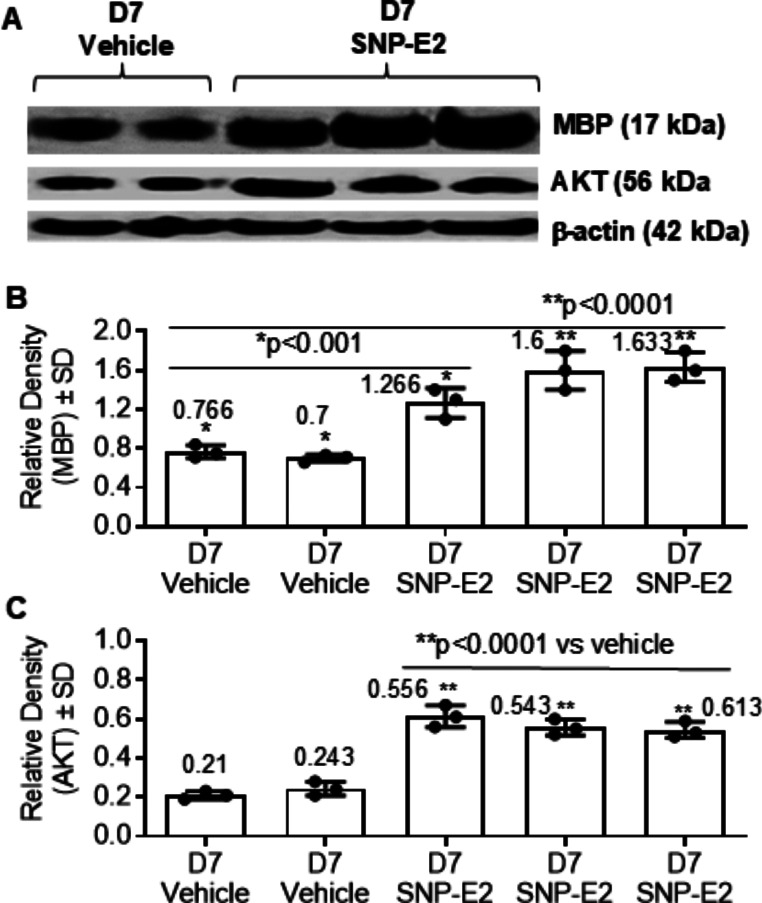
SNP-E2 therapy supports myelination and cell survival after SCI. Rats were treated with SNP-E2 immediately after SCI. Spinal cords (caudal sections) were collected 1 week after SCI. (**A**) Western bot analyses of spinal cord tissue were performed for determining MBP and AKT protein expression. (**B-C**) Protein band intensities for MBP and total AKT were analyzed via ImageJ software. *p < 0.05. *n* = 3–4

AKT regulates multiple cellular functions, including enhanced survival of oligodendrocytes (OLGs) and CNS myelination [49, 50]. Western blot analysis of caudal spinal cord sections showed that MBP and AKT expression levels were increased by focal delivery of SNP-E2. Data obtained suggest that SNP-E2 therapy promotes myelination after SCI in rats (Fig. 3, A-B). The expression of AKT was also increased, indicating that the unique aspects of SNP-E2 in enhancing survival signals (AKT) and protecting OLGs and myelin may have important implications for understanding remyelination and axonal protection in SCI.

### SNP-E2 Therapy Reduces Type 1 Microglial Activation and Promotes Differentiation of Type II Microglia in the Injured Spinal Cord

**Fig. 4:**
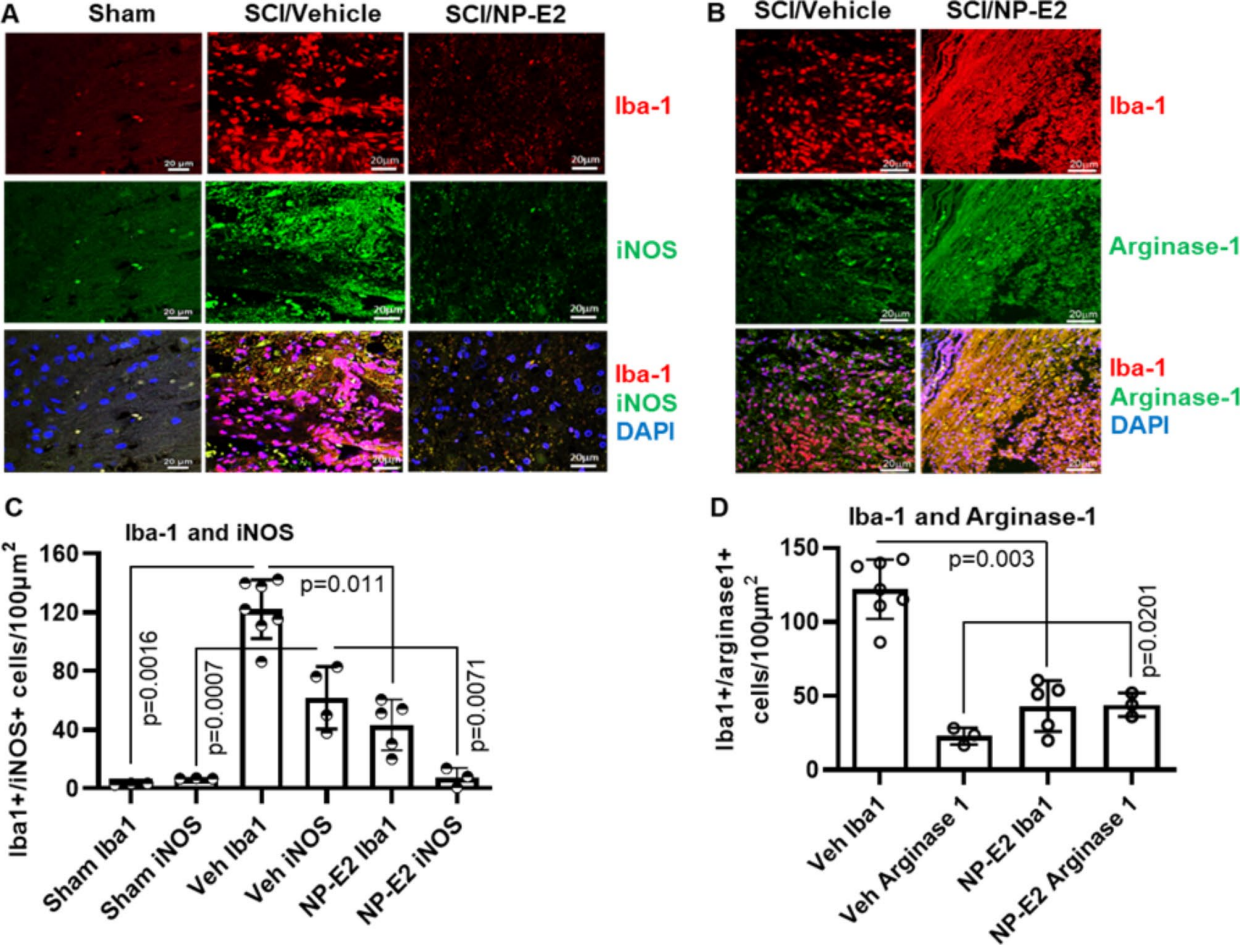
Focal delivery of SNP-E2 promotes differentiation of type II microglia in the injured spinal cord. (**A**) Spinal cord lesion sections were stained with either Iba-1 (red) or iNOS (green). (**B**) Sections were also stained with either Iba-1 (red) or Arginase-1 (green). DAPI was used for nuclear staining. (**C-D**) Quantitative analyses were performed by using ImageJ and GraphPad. Magnification, 20x. *n* = 3–5

Spinal cord sections caudal to the lesion (2 weeks and 4 weeks) from control and SNP-E2 treated rats were stained with Iba-1, iNOS, and arginase-1, and analyzed by confocal microscopy. Microglial activation especially iNOS-positive microglia (Iba-1) were pronounced in vehicle treated injured spinal cord as compared to sham (2.913 vs. 61.7), and they were decreased by NP-E2 treatment (61.7 vs. 7.366) Fig. 4. An increased number of arginase-1-positive microglia was detected in SNP-E2 treated spinal cord (22.903 vs. 44.13), suggesting that SNP-E2 therapy may promote M2 microglial differentiation following SCI. Overall, these data suggest that SNP-E2 gel patch therapy attenuates iNOS producing microglia (type 1) and supports the expansion of arginase-1/Iba-1 (type II) populations.

### SNP-E2 Therapy Promotes Maturation of OPCs in Severe SCI

**Fig. 5:**
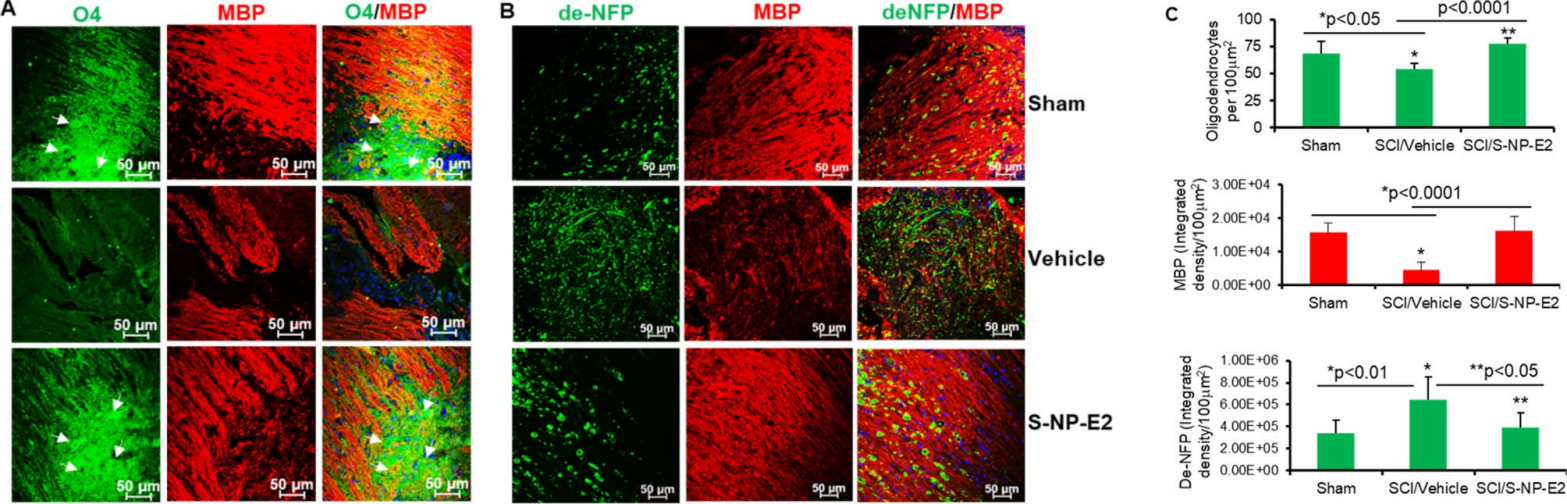
Focal delivery of SNP-E2 supports maturation of OPCs and induces protection of axons and myelin following severe (60gcf) SCI in rats. (**A**) Left panel, spinal cord lesion longitudinal sections were stained with oligodendrocyte marker O4 (green) and myelin basic protein MBP (red) and analyzed by confocal microscopy. (**B**) Right panel, confocal microscopic images of lesion sections at day 14 shows axonal damage (de-NFP, green) and demyelination (red) in vehicle group, and an improved myelination and less axonal damage after SNP-E2 therapy. Magnification, 20x. (**C**) Quantitative analysis of O4, MBP, and de-NFP. *n* = 3–4

To investigate whether SNP-E2 therapy supports OPCs in severe SCI (60 gcf), spinal cord sections (2 weeks after SCI) from sham control, injury vehicle, and SNP-E2 treated rats were stained with antibodies against oligodendrocyte (O4), myelin basic protein (MBP), and de-NFP (axonal damage). Axonal damage and OPC loss were observed in severely injured spinal cord sections, and those damages were attenuated by SNP-E2 therapy (Fig. 5). Loss of oligodendrocytes and MBP, as well as degeneration of axons are also evident in vehicle treated samples compared to sham while these losses are attenuated following treatment with SNP-E2 (Fig. 5, A, B, and C). These studies suggest that the focal delivery of E2 could be beneficial in protecting oligodendrocytes, myelin, and axons.

### Astrocyte-Mediated Functional Recovery in SCI Rats Following SNP-E2 Treatment

**Fig. 6:**
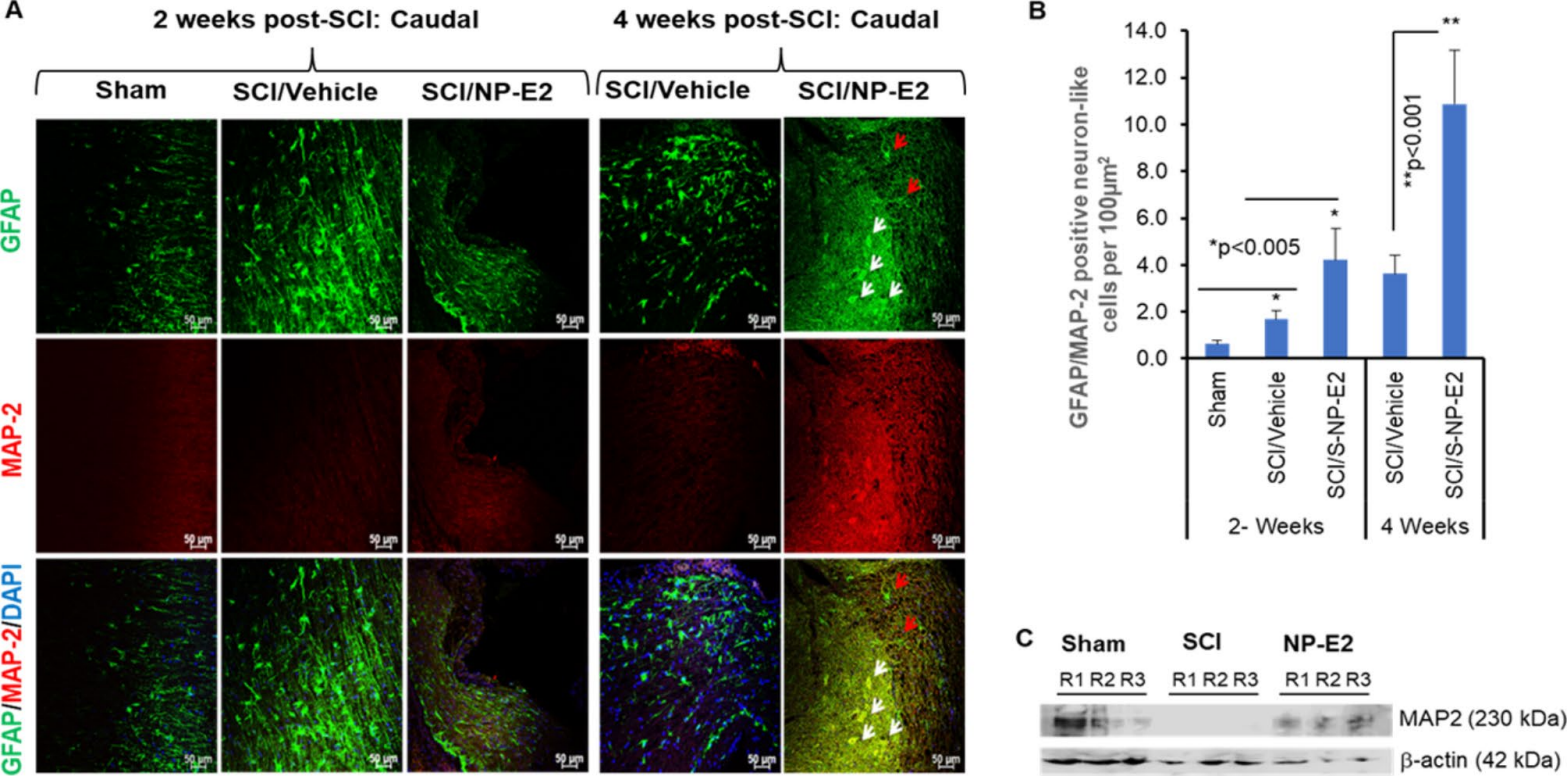
SNP-E2 therapy induces differentiation of GFAP-positive astrocytes to dual positive (MAP-2/GFAP) neuron like cells, promoting recovery after SCI. (**A**) Immunostaining of GFAP (green) and MAP-2 (red) in spinal cord tissues from untreated and SNP-E2 treated SCI rats. GFAP + MAP-2 + DAPI indicates overlay with DAPI. Magnification 20x. (**B**) Quantitative analysis of MAP-2/GFAP positive cells by ImageJ software. (**C**) Western blot analysis of spinal cord samples from 3 rats (R = rat) confirmed the generation of MAP-2 + neuron like cells in spinal cord after SNP-E2 treatment, suggesting a possible role of differentiated astrocytes in the neuronal repair process after SCI. *p < 0.05, *n* = 3–4

While SCI may lead to irreversible neuronal loss and glial scar formation, cellular regeneration can occur, and such evidence has recently been shown [50, 51]. Sustained release of E2 may help differentiate astrocytes to neuron-like cells, which could be a potential strategy for cellular differentiation and regeneration after SCI. Thus, in our experiments, IHC and confocal microscopic analyses of MAP-2 and GFAP markers in the spinal cord sections showed that contrary to early time-point (day 14) studies, increased expression of MAP-2 and GFAP is observed in the white matter of injured spinal cord at day 28 post-injury. MAP-2 expression was also observed in neurons of lesioned rats, but the intensity and extent were much lower compared to the injured spinal cord treated with SNP-E2 (Fig. 6A). Quantitative analyses of astrocytes and larger double positive (MAP-2/GFAP) neuron-like cells suggest that MAP-2+/GFAP + cells were significantly increased after SNP-E2 therapy (Fig. 6B). Understanding the role of neuron-like astrocytes after NP-E2 therapy may yield new insights into subtypes of astrocytes involved in functional recovery after SCI.

### SNP-E2 Improved Bladder and Locomotor Function

**Fig. 7:**
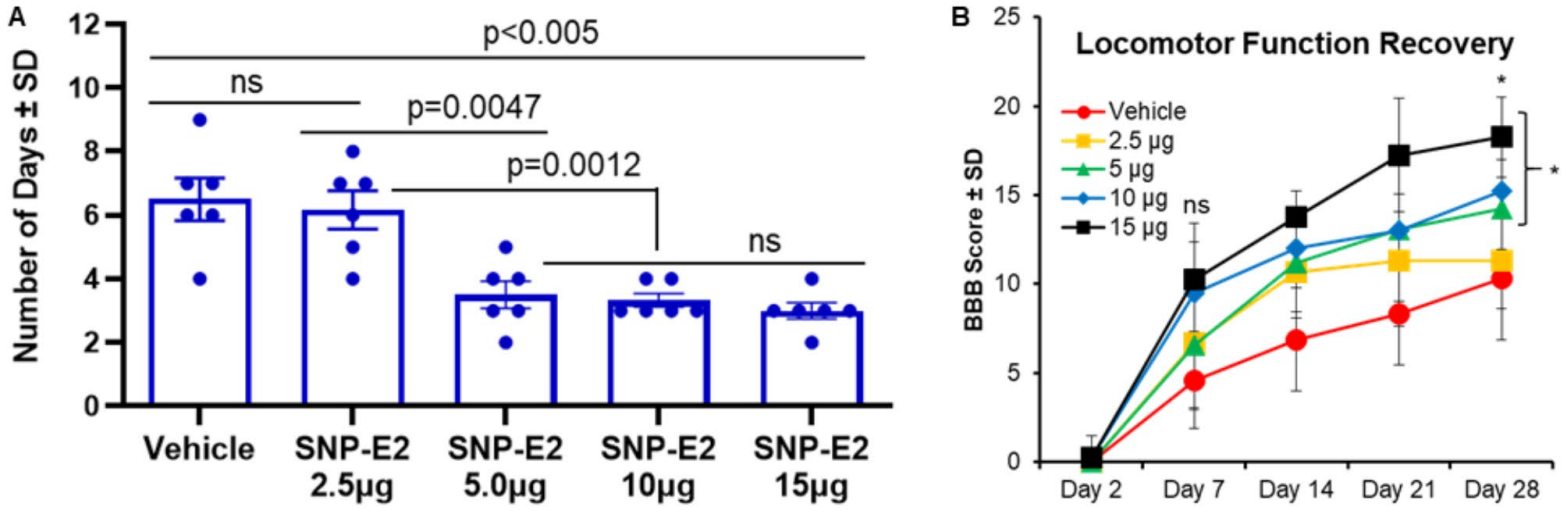
Bladder and BBB Assessment following SCI. (**A**) Bladder function recovery was assessed by monitoring the number of days until animals regained spontaneous bladder voiding. 5–15 µg of E2 treatment significantly decreased the number of days. *N* = 6. (**B**) Significant improvement in BBB score in the 5, 10, and 15 µg SNP-E2 group, as compared with vehicle and 2.5 µg on Day 21 and Day 28. Significant improvement in BBB score is also seen in 15 µg treatment group after Day 14. Sham animals, by criteria, are all scored a 21 at each time point and are therefore not displayed. *p < 0.05, vehicle versus SNP-E2 treatment groups. ns = not significant. *N* = 6–10

**Fig. 8:**
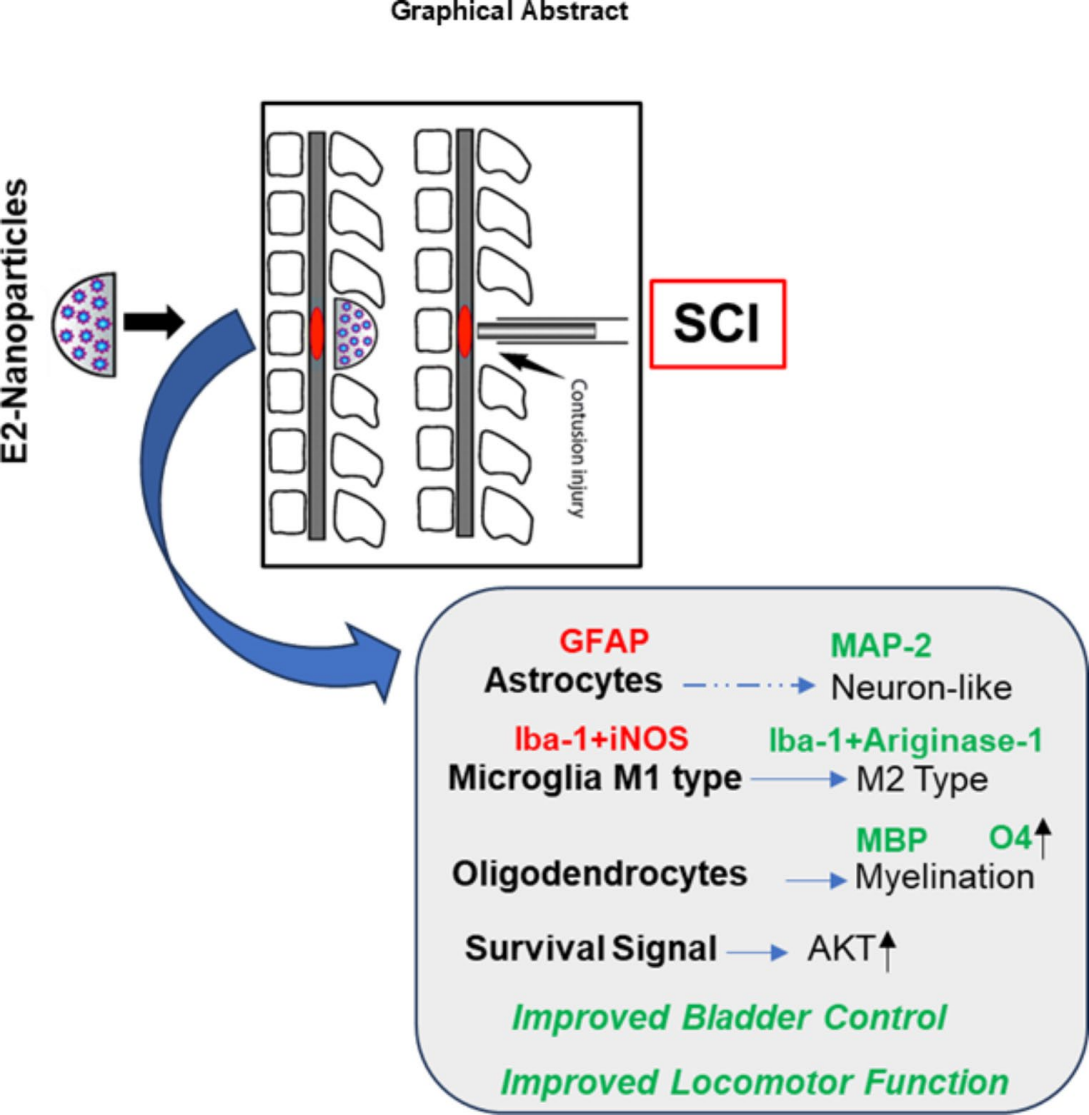
Graphical representation of findings from a rat model of SCI and slow-release E2-nanoparticle (SNP-E2) treatment. SCI induction was performed in the thoracic T10 region as described in the methods. A group of rats with SCI received SNP-E2, whereas the vehicle treatment groups received void nanoparticles. Sham rats had a T10 laminectomy. Spinal cord samples analyzed with immunohistochemistry and western blot indicated reduced inflammation and altered activated phenotypes of astroglia and microglia by SNP-E2 therapy, and a striking differentiation of astroglia to neuron-like cells. Additionally, increased myelination and neuronal survival were also detected in SNP-E2 treated rats. These significant changes, which underscore the importance of SNP-E2 treatment, are also accompanied by improved bladder control and locomotor function

SCI can cause changes in bladder function, known as neurogenic bladder, due to interference with nerve messages between the brain and bladder muscles [52, 53]. Thus, bladder function was tracked to examine a second functional endpoint in addition to motor function. SCI animals were first treated with 2.5–15 µg doses of E2 via SNP as indicated in the methods and bladder function was tested. Bladder function recovery was defined here as the ability to spontaneously void the bladder. The day in which this behavior was restored indicated that the animal no longer needed to be manually expressed. Data showed improved bladder function with the treatment of 5.0–15 µg dose after SCI (Figs. 7A and 8), suggesting that SNP-E2 attenuated neuronal injury in SCI.

Locomotor functional recovery was then investigated using the DigiGait treadmill system as described in the methods. Doses greater than 5.0 µg of E2 in SNP-E2 induced significant recovery of function after day 14 (Fig. 7B) as analyzed by one-way ANOVA. Overall, these data suggest that SNP-E2 therapy may have high translational potential as it reduces reactive gliosis in the early stages of the injury, improves myelination, protects neurons, and improves bladder and locomotor functions, as stated in the graphical representation (Fig. 8).

## Discussion

We have recently shown that FNP-E2 therapy significantly decreases post-SCI lesion size, reactive gliosis, and glial scar formation, suggesting that axonal regeneration, vascular endothelial growth factor production and glial-cell- derived neurotrophic factor levels could be increased with FNP-E2 administration [29]. These findings correlated with improved locomotor and bladder function in a mild to moderate SCI rat contusion model. Reducing the severity of both acute and chronic stages of SCI in a severe (60gcf) SCI model is harder than mild to moderate SCI. Thus, we have designed SNP-E2 and the delivery of this novel construct to the lesioned spinal cord resulted in E2 availability to the surrounding lesion area with no significant plasma E2 levels in SCI rats. The kinetics distribution in SCI rats suggested that the strategy is a viable approach for focal delivery of E2 for reducing inflammation and, more significantly, estrogenic toxicity. SNP-E2 prevented damage to oligodendrocytes, suggesting that inflammatory factors may have the potential to induce OLG death, which could promote demyelination. The proposed SNP-E2 treatment of severe SCI may produce better effects with negligible toxicity. While E2 is known to be a neuroprotectant, attempts to translate this promising therapeutic into the clinic have not been successful. Safety concerns associated with E2 intravenous (i.v.) dosing have thus far precluded clinical trial evaluation [34]. By using a focal delivery technique such as NPs, these safety concerns may be alleviated. Additionally, NP-E2 may be delivered for a prolonged period of time, particularly with the use of SNP-E2, allowing for extended bioavailability, enhanced regeneration, and functional improvement. Thus, the site-directed delivery of SNP-E2 may have significant translational value and clinical application not only in acute but also in chronic SCI.

Our present study suggests that SNP-E2 therapy either preserves or activates AKT in the injured spinal cord. The PI3K-AKT pathway is a key regulator of cell survival during cellular damage after SCI [54, 55]. Thus, the activation of AKT or restoration of AKT after SCI is important for neuronal survival and considered better therapeutic strategy for SCI. It is also considered a key regulator of autophagy due to its energy-sensing function and protective effects on glia and neurons as suggested [54, 56]. Preservation of AKT pathway also promotes motor function recovery in SCI rats. SCI causes neuronal death, axonal injury, and secondary tissue disruption [57, 58]. Demyelinated axons and axonal degeneration were observed in untreated SCI rats which were reduced following SNP-E2 treatment. Loss of myelin leaves axons vulnerable to degradation, further exacerbating functional deficits [57, 59]. Thus, reducing axonal degeneration, demyelination, and neural death could have a protective effect in the injured spinal cord. The extent of axon damage is an important predictor of clinical outcome following SCI and the number of axons that survive the injury makes a significant difference in locomotor functional outcome. Thus, how axonal recovery could be promoted is an important question in devising a comprehensive treatment strategy aimed at preserving and restoring spinal function. Our data showed that axonal damage was minimized by SNP-E2 treatment, suggesting our strategy has a translational potential in human SCI.

While homeostatic microglia are the predominant phenotype in the uninjured spinal cord after SCI, microglia become activated and produce inflammatory cytokines and chemokines [60, 61]. Our study suggests that proinflammatory microglia, especially type 1 microglia, are increased following SCI. These microglia produce iNOS and may play a role in inducing gliosis and the formation of an astrocytic scar that can contribute to axonal damage and neuronal death after SCI. Our data also suggests that SNP-E2 treatment induces differentiation of microglia into type 2 which produce arginase-1 and maybe they are anti-inflammatory. Increased presence of mature OPC by SNP-E2 therapy suggests that anti-inflammatory microglia (M2 type) could regulate oligodendrocyte survival, maintenance, and myelination in the injured spinal cord. M2 microglia are associated with anti-inflammatory responses and could reduce glial scar formation, protecting neurons. Our previous study with FNP-E2 treatment reduced glial scar formation and protected neurons in SCI rat model [29]. However, we have not tested in vivo effects of SNP-E2 on glial scarring in SCI yet, but our future study will examine whether SNP-E2 affects glial scarring following the induction of microglial polarization to the M2 phenotype in the optimized rat SCI model.

A gradual transformation of microglia from proinflammatory to an anti-inflammatory state may have occurred following SNP-E2 therapy that contributed to astrocytic differentiation or transformation. Detection of larger double positive (MAP-2+/GFAP+) neuron-like cells in the SNP-E2 treated injured spinal cord suggests that neural regeneration may lead to functional recovery in SCI rats. However, the specific mechanism required for these transformational changes of proliferated astrocytes to neuron-like cells needs to be further investigated.

Failure to recover motor function following SCI is due in part to inhibition of axonal regeneration and remyelination with the loss of neurons and OLGs. Understanding the mechanisms by which SNP-E2 therapy may address both acute and subacute phases of SCI by reducing glial and immune activation, protecting neurons, and improving function. Estrogenic effects are seen in glial cells and OPCs, which provides rationale for examining how long-term exposure to E2 may alter glial scarring and concomitant neuronal survival. Activated microglia/astrocytes produce a number of inflammatory factors, but the specific influence of microglia on OLGs as well as on OLG precursor cells (OPCs) is important [60–62]. Reactive astrocytes are capable of producing a variety of pro-inflammatory mediators and potentially neurotoxic compounds, including nitric oxide (NO). High amounts of NO are synthesized following up-regulation of inducible NO synthase (iNOS) [63, 64], which is expressed in the CNS in a signal-dependent fashion. Our study suggests a role for SNP-E2 treatment on microglial differentiation. The polarization of M1/M2 microglia after SCI, and their roles in so-called astrocytic polarization of neuron-like cells are important in neural survival. These findings indicate that the focal delivery of SNP-E2 may be beneficial in protecting oligodendrocytes, myelin, and axons. Along that line, SNP-E2 therapy supported a role for sustained release of E2 in inducing astroglial differentiation into neuron-like cells.

Astrocytes are the major glial cells present in CNS, and they outnumber neurons, microglia, or oligodendrocytes [65]. Reactive astrocytes express higher levels of iNOS, and they induce iNOS-mediated damage in the injured spinal cord [65, 66]. IL-1β and IFNγ can also induce iNOS in rodent astrocytes and it’s been shown that the activation of JAK2 could be involved in IFNγ-induced expression of iNOS in rat astrocytes [63, 64]. ROS are also known to regulate iNOS expression via NF-κB. This study found that gliosis and the production of iNOS is crucial for microglial differentiation into type 2 which may favor astrocytic conversion of MAP2/NeuN type cells. Astrocytes are the most abundant cell type in adult neural tissue and are indispensable for maintaining normal health and function of the CNS [65, 67]. In response to SCI, astrocyte activation occurs, accompanied by changes in microglial activation, altered gene expression, and consequent morphology and functional changes in these cells. Reactive astrocytes are common in SCI and play inflammatory roles in inducing axonal damage and neuronal death. However, astrocytic conversion into so-called A2 astrocytes may play a role in inhibiting neuroinflammation and inducing neuroprotection in the CNS [68]. Thus, the heterogeneity of reactive astrocytes needs to be investigated in a setting of neurotrauma and our study with SNP-E2 may allow us to further investigate astrocytic differentiation and neural regeneration in SCI as suggested by others [69].

While OPCs contribute to remyelination by oligodendrogenesis, the functions of OPCs need to be restored by blocking glial scar formation. Our earlier study suggests that delivery of E2 decreases post-injury lesion size, reactive gliosis, and glial scar formation [29]. The current study showed that SNP-E2 mediated reduction of gliosis and microglial differentiation into M2 type protected OPCs and improved myelination. The extent of axon damage is an important predictor of clinical outcome following SCI and the number of axons that survive the injury makes a significant difference in functional outcome [34, 70]. Thus, how axonal recovery could be promoted is an important question in devising a comprehensive treatment strategy aimed at preserving and restoring spinal function. Our data showed that axonal damage was minimized by SNP-E2 treatment, suggesting our strategy has a translational potential in human SCI. Coordinated function between the bladder and urethral sphincter is disrupted after SCI, and the degree of dyssynergia is related to the severity of spinal injury. This loss of coordination leads to functional bladder outlet obstruction identified by urinary retention and increased micturition pressure. Non-voiding contractions manifested as phasic bladder contractions during urine storage, result in urinary incontinence and high intravesical pressures, leading to bladder hypertrophy and deterioration of the upper urinary tract. Neurogenic bladder dysfunction is also a condition that affects both bladder storage and voiding function and remains one of the leading causes of morbidity after SCI. Animal models are critical to our fundamental understanding of lower urinary tract function and its dysfunction after SCI, in addition to providing a platform for the assessment of potential therapies.

BBB locomotor rating scale has been known to be a valid and predictive measure of locomotor recovery. Our current study found mean BBB scores in the SNP-E2 treated SCI group were higher than those in the vehicle treatment group reaching statistical significance at 5–15 g of E2 in the SNP-E2 construct. Neurogenic bladder dysfunction has been correlated with SCI severity [29, 71]. On the other hand, improved locomotor function is also associated with increased bladder capacity and enhanced perception of bladder filling. Our data showed that SNP-E2 therapy benefitted both bladder function and locomotor function in SCI rats, indicating that the treatment strategy decreased inflammation at the cellular level and improved functional outcome after SCI. In conclusion, our findings suggest that the focal delivery of SNP-E2 has a significant therapeutic potential for treatment of SCI individuals.

## Data Availability

No datasets were generated or analysed during the current study.
